# Decisional conflict in patients considering diagnostic thyroidectomy with indeterminate fine needle aspirate cytopathology

**DOI:** 10.1186/s40463-016-0130-x

**Published:** 2016-02-27

**Authors:** Benjamin A. Taylor, Robert D. Hart, Matthew H. Rigby, Jonathan Trites, S. Mark Taylor, Paul Hong

**Affiliations:** Division of Otolaryngology Head and Neck Surgery, Department of Surgery, IWK Health Centre, Dalhousie University, 5850 University Avenue, Halifax, NS B3K 6R8 Canada

**Keywords:** Decisional conflict, Thyroid cancer, Thyroidectomy, Fine needle aspiration, Shared decision-making

## Abstract

**Background:**

Fine needle aspiration (FNA) cytopathology is the gold standard work-up for thyroid nodules. However, indeterminate lesions are encountered commonly and can lead to difficult treatment decisions. We sought to determine whether patients experienced decisional conflict surrounding management with diagnostic thyroidectomy in the setting of indeterminate FNA results.

**Methods:**

Patients with indeterminate results of thyroid nodule FNA were prospectively enrolled. All consultations were carried out by three otolaryngologists in a consistent manner. After consultation, participants completed a demographics form and the Decisional Conflict Scale (DCS) questionnaire.

**Results:**

Thirty-five patients (28 female) between the ages of 30 and 88 years (mean age 54.89) participated. The median total DCS score was 10.94 (interquartile range, 4.69–25.0). Twelve patients (34 %) scored at or above 25 on the DCS, indicating clinically significant level of decisional conflict. Patients reported feeling significantly more confident about their decision after the surgical consultation compared to before the consultation (*p* = 0.00). The total DCS score was significantly negatively correlated with self-reported confidence after the consultation (*r* = −0.421, *p* = 0.012).

**Conclusion:**

Many patients experienced clinically significant decisional conflict when considering thyroidectomy for management of a thyroid nodule with indeterminate cytopathology. Future research should be directed at developing decision support tools for this patient group, and exploring the impact of decisional conflict on health outcomes.

## Background

Thyroid cancer has demonstrated the most rapid increase in incidence of any cancer in North America, and now ranks as the fifth most common cancer in women [1, 2]. Part of the increase is attributed to early detection, often incidentally, through improved diagnostic imaging techniques [3–5].

One of the most important diagnostic tools for suspected thyroid cancer is the fine needle aspirate (FNA). The FNA is performed for most thyroid nodules greater than 1.5 cm, or smaller in patients with suspicious sonographic features or those with high-risk history [6]. Over 500,000 thyroid nodule FNA procedures are performed annually in the United States [7]. The sample obtained via FNA is analyzed cytopathologically and reported at most institutions with the guidance of the Bethesda Grading System [8]. The Bethesda system describes six categories: nondiagnostic or unsatisfactory, benign, atypia of undetermined significance (AUS) or follicular lesion of undetermined significance (FLUS), follicular neoplasm or suspicious for a follicular neoplasm (SFN), suspicious for malignancy (SFM), or malignant [8]. Indeterminate results include AUS, FLUS, and SFN, which are encountered in about 15–30 % of samples, and carry a 6–32 % risk of malignancy [7, 9, 10]. Practice guidelines have traditionally recommended surgery for indeterminate lesions; however, final pathology yields a benign result in 70–85 % of cases [7].

Patients with indeterminate results on FNA who are considering diagnostic thyroidectomy may face challenges in decision-making because of limited consultation time with surgeons, complexity of information on risks/benefits, and the uncertainty of their diagnosis. As a result, patients may experience *decisional conflict*, which can lead to emotional distress and other negative sequelae [11, 12]. Tools such as decision aids can help patients and practitioners become more involved in decision-making by providing information about treatment options and outcomes, clarifying personal values about the treatment, and providing guidance throughout the decision-making process. The use of decision aids has been shown to result in a range of favorable outcomes including less decisional conflict, improved patient knowledge, and greater concordance between patient values and chosen treatment option [13]. As well, the use of decision aids can lead to reduction in unnecessary variation in care and costs across different healthcare regions [14]. In fact, there is a sleeper provision in the Affordable Care Act (Section 3506) that encourages the use of shared decision-making in healthcare with decision aids. Before a decision aid can be appropriately developed, a decision-needs analysis is first required, which includes measuring the level of decisional conflict in certain procedures.

There is a paucity of data in the literature surrounding decisional conflict and to date, none are available for diagnostic thyroidectomy. The purpose of this study was to assess the level of decisional conflict in patients with an indeterminate result on thyroid nodule FNA who are considering thyroidectomy. We also evaluated the relationship between patient factors and decisional conflict

## Methods

All adult patients who received an indeterminate result on thyroid FNA during the study period (January 2014 to June 2014) were invited to participate in this study. The only exclusion criteria was the inability to speak or read English (*n* = 0) or the lack of decision-making authority (*n* = 0). Also, patients who had multiple FNAs of the same thyroid nodule were also excluded (*n* = 4). All patients were being considered for diagnostic hemi-thyroidectomy.

Patients were approached after the consultation visit with their head and neck surgeon where they were informed of their indeterminate FNA results. Informed consent for inclusion in this study was obtained from all those who agreed to participate. Each patient was asked to complete a Demographic/Condition form and the Decisional Conflict Scale (DCS).

For providers, three head and neck fellowship trained otolaryngologists participated. All used a consistent script to ensure that similar information was shared during the visit.

Local Institutional Review Board approval was obtained for this study.

### Demographic/condition form

The demographic information collected on this form included patient age, family composition, employment status, occupation and income. Data was also collected on previous surgical experience, confidence level in their medical decision-making before and after the consultation, how well they usually handle medical appointments, and whether they were aware that surgery was an option before presenting.

### Decisional conflict scale (DCS)

This 16-item scale assesses patient uncertainty about medical decisions. It is a Likert scale with ratings of strongly agree, agree, neither agree or disagree, disagree, and strongly disagree. The DCS produces a total score ranging from 0 (no decisional conflict) to 100 (maximal decisional conflict). Five Subscales scores are also produced. The subscales are interpreted as follows: uncertainty subscale [scores range from 0 (extremely certain about best choice) to 100 (extremely uncertain about best choice)], informed subscale [0 (feels extremely informed) to 100 (feels extremely uninformed)], values clarity subscale [0 (feels extremely clear about personal values for benefits and risks) to 100 (feels extremely unclear about personal values)], support subscale [0 (extremely supported in decision making) to 100 (extremely unsupported in decision making)], and effective decision-making subscale [0 (good decision) to 100 (bad decision)] [16].

The DCS is a validated scale that is designed to be context non-specific. It has demonstrated high test-retest reliability and high content validity, as scores on the DCS were higher for patients who delayed or were unsure of their decision in comparison to those who accepted or rejected treatments [16]. Previous research has defined clinically significant decisional conflict as a DCS score at or above 25 [16].

### Data analysis

The DCS scores were not normally distributed; therefore, nonparametric statistical tests were used. Descriptive statistics including median, interquartile range (IQR), and standard error (SE) of the total DCS scores are reported. The DCS subscale scores along with the number of patients with total DCS score at or above 25, indicating clinically significant decisional conflict, are also reported. The relationship between baseline factors and total DCS scores were explored using Mann Whitney U tests.

## Results

### Participants

The study was conducted at a head and neck oncology clinic situated in a tertiary care academic hospital in Eastern Canada. Thirty-five new consecutive patients who met the inclusion criteria were invited to participate in this study. All patients approached agreed to participate. None of the patients had other diagnostic testing (e.g., molecular testing or gene profile analysis).

Patients ranged in age from 30 to 88 years (mean = 54.89, SD = 15.30). Twenty-eight (80 %) patients were female and 7 (20 %) were male. Twenty-eight (80 %) patients had undergone previous surgery in the past, and 19 (68 %) of those had undergone more than one. The most common operations reported were tonsillectomy (*n* = 7), gynecological procedures (*n* = 6), cesarean section (*n* = 5), breast surgery (*n* = 5), and cholecystectomy (*n* = 4). Twenty (57 %) patients had family members who had undergone previous surgery. All patients reported that they had handled previous medical visits well (*n* = 9) or very well (*n* = 26). Most patients (*n* = 33, 94 %) reported that they knew surgery was an option prior to the consultation visit, while one patient was unaware (data was unavailable for one participant).

Three head and neck fellowship trained otolaryngologists, ranging in age from 36 to 46 years, participated. All were male. All were in a salaried academic practice and all trained in North America.

### Decisional conflict

**Fig. 1:**
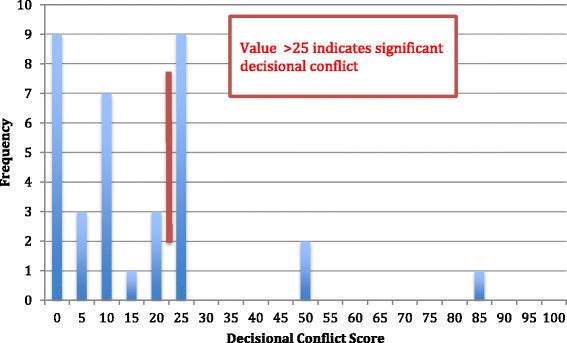
Frequency of total Decisional Conflict Scale scores. Scores to the right of the red line (≥25) indicate clinically significant decisional conflict

The median total DCS score was 10.94 (SE = 3.01, IQR = 4.69–25). Twelve patients (34 %) were found to have clinically significant decisional conflict, as they scored at or above 25 on the DCS (Fig. 1). Three patients (8.5 %) scored zero on the DCS, indicating no uncertainty about their decision. Summary of the DCS results is presented in Table 1.

**Table 1:** Median total decisional conflict scale and subscale scores

	Total	Uncertainty	Informed	Clarity	Support	Effective
Median	10.94	25.00	8.33	25.00	8.33	12.50
SE	3.01	3.23	2.32	2.57	2.08	2.27
IQR	4.69–25	8.33–25	0.00–25	0.00–25	0.00–25	0.00–25

*Abbreviations*: *SE* standard error, *IQR* interquartile range

There were no significant differences in the total DCS scores or subscale scores based on whether the patient had previous surgery or if another family member had previous surgery. As well, no significant correlation existed between DCS scores and patient income, patient gender, or previous awareness of surgery. However, there was a significantly negative correlation between values clarity subscale scores and patients’ self-report of how well they tolerated previous surgery (*r* = −0.347, *p* = 0.041). As well, patient age and support subscale scores were significantly positively correlated (*r* = 0.382, *p* = 0.023).

Patients’ self-reported confidence levels were significantly higher after surgical consultation (mean = 7.11 out of 10) compared to before consultation (mean = 6.43 out of 10; *p* = 0.00). The total DCS score was significantly negatively correlated with confidence level after consultation (*r* = −0.421, *p* = 0.012).

## Discussion

It is estimated that 15–30 % of FNA samples yield indeterminate results, of which 70–85 % are diagnosed to be benign after thyroidectomy [3, 4]. In light of this uncertainty, patients may experience decisional conflict when faced with the decision to proceed with diagnostic surgery. In the current study, the median total DCS score was 10.94. However, 12 (34 %) individuals scored at or above the cutoff score of 25, indicating the presence of clinically significant decisional conflict. This level of decisional conflict is similar to previous studies that have assessed other elective surgical procedures [12, 15, 18–23]. Given the high prevalence of indeterminate thyroid lesions, the number of patients experiencing decisional conflict may be substantial. Decisional conflict is associated with negative outcomes such as emotional distress, cancelled surgeries, and non-adherence to treatment plans [12]. To overcome this problem, an approach is needed to help both healthcare providers and their patients in surgical decision-making.

*Shared decision-making* is a strategy that could be used in the current patient population. This is an approach that requires collaboration between healthcare providers and their patients to understand the treatment options and have knowledge of the risks and benefits of these options. At the same time, shared decisions should consider the patients’ own preferences and values in decision-making [17]. The importance of shared decision-making goes well beyond reducing decisional conflict and the associated negative outcomes. This includes improved quality of care and reduced variation in care and costs across different regions since there is a more consistent decision-making process and better compliance with clinical guidelines [15]. As mentioned above, there is a provision in the Affordable Care Act that encourages the use of shared decision-making in healthcare [24]. Unfortunately, the concept of shared decision-making in medicine is in its infancy at this time.

To date, no studies have assessed the level of decisional conflict in patients considering diagnostic thyroidectomy. However, a decisional conflict analysis was performed on patients considering adjuvant radioactive iodine treatment for early stage papillary thyroid cancer as part of a randomized control trial assessing the utility of a decision aid. The mean total DCS score was very high (52.1, SD = 21.9) in those patients deciding on whether to accept or reject the radioactive iodine treatment [18]. Clearly, significant decisional conflict is prevalent in patients who are undergoing work-up and treatment of thyroid nodules and malignancies.

There are other studies reporting decisional conflict in patients undergoing work-up and treatment of malignancies. Women diagnosed with ductal carcinoma in situ of the breast were found to have a mean total DCS score of 20.5 with 47 % reporting clinically significant decisional conflict when considering various treatment options [19]. Another study with breast cancer patients faced with surgical decisions reported a mean total DCS score of 19.9 [20]. Studies in patients dealing with prostate cancer screening and pre-treatment decisions found a mean total DCS score of 25 [21] and 53 [22], respectively. Again, these data indicate that decisional conflict is common in patients with common malignancies.

Subscale analysis of the DCS showed that the uncertainty and values clarity subscales had the highest levels of decisional conflict. The informed and support subscales had the lowest levels of decisional conflict. Patient age and support subscale scores were significantly positively correlated, indicating that older patients were more likely to have less support in their decision-making. This is an important finding as older patients without adequate social or family support may be at higher risk of having significant decisional conflict. Therefore, clinicians may need to provide additional decision support to some older patients.

A significant negative correlation existed between values clarity subscale scores and patients’ self-report on how well they handled previous surgeries. This indicates that patients were more likely to feel clearer about personal values for the benefits and risks of surgery if they did not handle previous operations well. One would expect that a patient with positive previous surgical experience would be more familiar and perhaps more insightful to their personal values regarding surgery. However, the converse was noted in the current study. The explanation for this finding is unclear but the modest nature of the correlation should be iterated (*r* = −0.347, *p* = 0.041).

Patients were significantly more confident in their decision-making after the surgical consultation, compared to before. This was a reassuring finding that consultation with surgeons, which involved a detailed discussion of the risks and benefits of the diagnostic surgery, had a positive effect on the decision-making process. However, it is to be noted that the change in confidence level (6.43 to 7.11 out of 10) was small and the clinical significance remains unclear. Therefore, effect size calculation with increased sample size is required to make a more definitive conclusion.

Unsurprisingly, the total DCS score was significantly negatively correlated with self-reported confidence level after the consultation visit. Although causation cannot be proven, this suggests that most individuals felt more confident after the consultation, which may have contributed to lower levels of decisional conflict.

*Decision aids* are evidence-based tools used to support patients in challenging medical decision-making situations (i.e., when there isn’t one superior treatment option). Decision aids have been shown to improve patient knowledge, enhance shared decision-making, increase the number of patients with realistic ideas about the risks and benefits of a medical procedure, and reduce decisional conflict [23]. They can also empower the patient with information to facilitate a more meaningful and informed discussion with their physician [24]. Decision aids usually include three sections: 1) a description of the health condition and management options being considered; 2) a summary of the evidence for each of these options including risks and benefits; 3) and an element to help the patient consider this information in the context of their personal values [25].

In oncology, decision aids have been used in cancer patients considering surgical intervention with good success. In a meta-analysis, O’Brien et al. found that the use of decision aids in cancer related illness leads to significantly improved knowledge about screening, treatment, and preventative measures compared to usual practice [26]. Overall, the use of well-developed decision aids led to reduced decisional conflict and no increase in general anxiety [26]. Further support for decision aids comes from randomized controlled trials in patients with thyroid, breast, and prostate malignancies. Decisional conflict was significantly reduced in patients given decision aids, compared to those who underwent conventional consultation [18, 19, 21, 22]. Although there are many positive aspects, no comprehensive evidence-based decision aids exist in otolaryngology at this time [25]. Clearly, decision support tools, such as decision aids, would benefit patients with indeterminate thyroid nodule FNA results.

There has been a recent call for the development of decision aids in otolaryngology as there are many elective surgical procedures within this specialty (e.g., tonsillectomy, sinus surgery, rhinoplasty) [25, 27]. Although there are numerous processes reported for developing decision aids [28], the most rigorous and well-received method is best practices recommended by the International Patient Decision Aid Standards (IPDAS) Collaboration [29]. The IPDAS framework outlines an iterative process, which allows multiple stakeholders (e.g., patients, clinicians, decision experts) to define their needs so that the decision aid will be feasible and useful to all potential users. Even though there are a number of decision aids currently available, many have been developed without following a specific methodology, which can lead to poor quality and information presented in a biased manner [28]. Therefore, decision aids in otolaryngology and beyond should be created following the method outlined by the IPDAS Collaboration.

Limitations of this study include the involvement of multiple head and neck surgeons. This caused an inherent variability of approach to patients and counseling style, perhaps resulting in different experiences for patients. To control for this, the surgeons used a semi-structured interview script to keep the delivered information consistent (i.e., same risks and benefits discussed). Unfortunately, the sample size did not allow for direct statistical comparison of the DCS scores between surgeons. The use of multiple providers does, however, allow for a more broad perspective of the decisional conflict that exists across the patient population. The second limitation of this study is the consideration of only indeterminate thyroid lesions. More information regarding decisional conflict could be gleaned from comparison of all FNA results. Third, the long-term influence of clinically significant decisional conflict is unknown in our study population. That is, some patients with decisional conflict may have been less satisfied with their overall experience or may have changed their decision over time. Fourth, some demographic information was not measured (e.g., education levels, ethnicity) that may have influenced the level of post-consultation decisional conflict. Finally, the sample size of the participants was small, but was comparable to other studies assessing decisional conflict [12, 15, 18–22, 30].

To advance research in this area, future studies should consider incorporating observations of consultation visits (e.g., video-recording) of a larger number of providers across multiple healthcare centers. Moreover, research should assess long-term outcomes of decisional conflict including knowledge about the procedure and postoperative care. Finally, as mentioned above, future research should aim to identify strategies that could improve the decision making process for patients (and providers) with the potential aim of developing decision support tools, such as decision aids.

## Conclusion

Many patients with indeterminate thyroid FNA results experienced clinically significant decisional conflict and therefore were uncertain about their decision to proceed with diagnostic thyroidectomy. Decisional conflict has been associated with many negative outcomes, and therefore future research should aim to find methods to reduce decisional conflict.
